# Brain Infraslow Activity Correlates With Arousal Levels

**DOI:** 10.3389/fnins.2022.765585

**Published:** 2022-02-25

**Authors:** Duho Sihn, Sung-Phil Kim

**Affiliations:** Department of Biomedical Engineering, Ulsan National Institute of Science and Technology, Ulsan, South Korea

**Keywords:** infraslow activity, electroencephalogram, arousal, galvanic skin response, phase-amplitude coupling (PAC)

## Abstract

The functional role of the brain’s infraslow activity (ISA, 0.01–0.1 Hz) in human behavior has yet to be elucidated. To date, it has been shown that the brain’s ISA correlates with behavioral performance; task performance is more likely to increase when executed at a specific ISA phase. However, it is unclear how the ISA correlates behavioral performance. We hypothesized that the ISA phase correlation of behavioral performance is mediated by arousal. Our data analysis results showed that the electroencephalogram (EEG) ISA phase was correlated with the galvanic skin response (GSR) amplitude, a measure of the arousal level. Furthermore, subjects whose EEG ISA phase correlated with the GSR amplitude more strongly also showed greater EEG ISA modulation during meditation, which implies an intimate relationship between brain ISA and arousal. These results may help improve understanding of the functional role of the brain’s ISA.

## Introduction

Infraslow activity (ISA, 0.01–0.1 Hz) was first recorded electrophysiologically in the rabbit brain in the 1950s (Aladjalova, 1957). Since then, it has been observed with electroencephalography (EEG) in the human brain during waking (Monto et al., 2008; Karavaev et al., 2018; de Goede and van Putten, 2019) and during sleep (Marshall et al., 2000; Vanhatalo et al., 2004; Lázár et al., 2019). EEG ISA has been shown to correlate with blood-oxygen-level-dependent (BOLD) signals with some time lag (Pan et al., 2013; Grooms et al., 2017). Based on well-documented correlations between BOLD signals and behavioral performance (Fox et al., 2007), EEG ISA is also speculated to correlate with behavioral performance. Monto et al. (2008) reported that behavioral performance is enhanced at the specific oscillation phase of the EEG ISA. However, it remains unclear how the brain’s ISA phase correlates with behavioral performance.

Arousal levels have shown some association with modulation of behavioral performance. Behavioral performance decreases when arousal levels are too low or too high and are optimal at intermediate arousal levels (Yerkes and Dodson, 1908; McGinley et al., 2015; McCormick et al., 2020). We hypothesized that the brain ISA phase might correlate with behavioral performance, in part, through the correlation with arousal levels. To measure arousal levels, we used the galvanic skin response (GSR), the activity of the sympathetic nervous system measured on the skin (Neumann and Blanton, 1970; Venables and Christie, 1980), which is known to correlate with arousal levels.

To address this hypothesis, we analyzed the coupling between the EEG ISA phase and the GSR amplitude in humans during meditation. Since meditation affects the arousal level (Cuthbert et al., 1981; Zeier, 1984), we used data recorded during a repetitive meditation task to induce changes in arousal levels which could be correlated with EEG ISA, where each trial of the repetitive meditation task is a restart of meditation. It was expected that the arousal level would change with each trial as meditation was resumed. Hence, the data in this meditation task would suit well to the purpose of our study to investigate whether EEG ISA is coupled with the arousal level.

We found that the EEG ISA phase was coupled with the amplitude of the GSR in humans during meditation. Furthermore, subjects whose EEG ISA phase was coupled with the GSR amplitude more strongly showed greater EEG ISA modulation itself during meditation. This suggests an intrinsic relationship between brain ISA and arousal levels, which might relate to the observed correlation of behavioral performance with brain ISA.

## Materials and Methods

### Dataset

**FIGURE 1:**
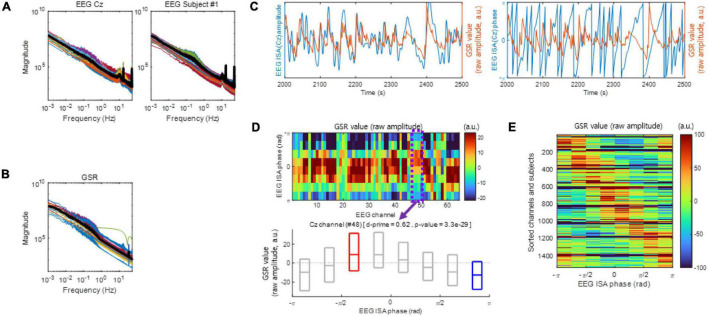
Electroencephalogram infraslow activity (ISA) and the galvanic skin response (GSR). **(A)** The EEG magnitude vs. frequency. Left: Each color-coded line indicates a different subject (left) or a different channel (right). The bold black line indicates the median. **(B)** same as **(A)**, but with the GSR magnitude plotted vs. frequency. **(C)** Examples of EEG ISA and the GSR over the period from 2,000 to 2,500 s which is an arbitrary period (Subject 1). Left panel exhibits amplitude and right panel exhibit phase. Notice that the peaks of the GSR magnitude appear to be in the rising phases of EEG ISA. **(D)** Examples of coupling between the EEG ISA phase and the GSR amplitude (Subject 1). (top) Distributions of the GSR amplitudes over the EEG ISA phase for different EEG channels. The GSR amplitudes appear to vary systematically with the EEG ISA phase in each channel. (bottom) An example of the distribution of the GSR amplitude over the EEG ISA phase at channel Cz (Subject 1). The boxplot indicates the 25, 50, and 75% of the GSR amplitude data. The d-prime and *p*-value were calculated by a comparison between the GSR amplitudes at the EEG ISA phase with the maximum median GSR amplitude (red box) and at the EEG ISA phase with the minimum median GSR amplitude (blue box). **(E)** Aggregate of panel **(D)** across subjects and channels. Note that the phase difference between the maximum GSR and the minimum is approximately π.

We used the dataset publicly available at https://openneuro.org/datasets/ds001787/versions/1.0.3 (Delorme and Brandmeyer, 2020). The dataset contains 64 channels of EEG data, GSR data, and respiration data recorded from 12 expert and 12 novice meditators performing meditation. The dataset was recorded using a DC coupled amplifier (64-channel Biosemi system), which allows DC measurements^1^ to enable the EEG analysis in the infraslow frequency band. Also, as shown in Figures 1A,B, the low-frequency components of EEG signals in this dataset are not attenuated, demonstrating that no highpass filtering with (>0.1 Hz) was applied to the raw EEG signals. A meditation task lasting 30–90 s was repeated with an intermittent pause in which the meditators answered questions about the quality of the meditation (Brandmeyer and Delorme, 2018). The average number of trials across subjects was 40.96 ± 12.53. The sampling rates of the EEG, GSR, and respiration data were 256 Hz.

### Data Processing and Analysis

To estimate the phase of the EEG ISA, EEG signals were Hilbert-transformed after being filtered by a bandpass filter at 0.01–0.1 Hz. Since common motion-related EEG artifacts such as eye-blinks typically contain frequency components faster (>1 Hz) than ISA frequency band, an appropriate bandpass filter (0.01–0.1 Hz) can filter out these motion-related EEG artifacts. Therefore, we did not apply further artifact removal processes after bandpass filtering. Such simplified preprocessing can be an additional merit for the development of real-time ISA-based applications. The instantaneous frequency of the EEG ISA was calculated from the Hilbert-transformed signals by calculating the derivative of the phase. The GSR and respiration signals were highpass-filtered with a cutoff frequency of 0.01 Hz to remove spurious drifts. After data processing (Hilbert transformation, instantaneous frequency calculation, and highpass filtering), all data were downsampled from 256 to 1 Hz.

To qualify the discriminability between two data, the d-prime was calculated as follows (Das and Geisler, 2021):


(1) $$d\,\text{-}\,\mathrm{prime}=\frac{\left|\mu_{1}-\mu_{2}\right|}{\left(\sigma_{1}+\sigma_{2}\right)/2}$$

where μ_*k*_ is the mean and σ_*k*_ is the standard deviation of the *k*th data, *k* = 1 or 2. *k* = 1 represents the GSR amplitude data at the EEG ISA phase with the maximum median GSR amplitude. *k* = 2 represents the GSR amplitude data at the EEG ISA phase with the minimum median GSR amplitude.

Inter-channel phase coherence (ICPC) or inter-trial phase coherence (ITPC) at time *t* after trial onset were calculated as follows (van Diepen and Mazaheri, 2018):


(2) $$I\,C\,P\,C=\left|\frac{1}{N}\,\sum_{n=1}^{N}\exp i\,\theta_{n}\right|$$

where θ_*n*_ is the EEG ISA phase estimated at the *n*th channel, and *N* is the total number of channels. On the other hand, ITPC was calculated as:


(3) $$I\,T\,P\,C_{t}=\left|\frac{1}{N}\,\sum_{n=1}^{N}\exp i\,\theta_{n,t}\right|$$

where θ_*n*,*t*_ is the EEG ISA phase estimated in the *n*th trial at time *t*, and *N* is the total number of trials. A trial here represents each run of the meditation task. The values of ICPC and ITPC range from 0 to 1. To examine the statistical significance of the ITPC values, a permutation test was performed. The test statistics for the permutation test were computed as follows: (1) θ_*n*,*t*_ was chosen from pseudo-random timing (one of the time points from the entire recordings) and pseudo-ITPC was calculated based on these θ_*n*,*t*_ of *N* samples; (2) step (1) was repeated 10^4^ times; and (3) the set of samples for test statistics was a collection of these pseudo-ITPCs.

For data analysis during meditation, each epoch was defined as the first 30-s period immediately after the onset of meditation. For frequency analysis, the frequency magnitudes were defined as the absolute values of the Fourier-transformed data. Fourier transforms were conducted using the fast Fourier transform (FFT) algorithm.

## Results

### The Phase of Electroencephalogram Infraslow Activity Is Coupled With the Amplitude of the Galvanic Skin Response

In Monto et al. (2008), the frequency–magnitude relationship of the EEG ISA showed log–log linearity. Similarly, in the present study, both EEG ISA (Figure 1A) and the GSR (Figure 1B) showed log–log linearity between frequency and magnitude without clear peaks, which was reminiscent of 1/f noise. However, the phase of the EEG ISA was coupled with the amplitude of the GSR (Figures 1C,D), indicating that the EEG ISA was different from noise.

To analyze the coupling between the EEG ISA phase and the GSR amplitude, we divided the EEG ISA phases into eight bins (from −π to −π with a step of π/4 in radians) and measured the average GSR amplitude within each bin for each EEG channel. To examine the significance of the coupling between the EEG ISA phase and the GSR amplitude, we identified the EEG ISA phase with the maximum GSR activity and that with the minimum GSR activity for each EEG channel. The mean phase differences between the two indexed EEG ISA phases were nearly π (Figure 1E), indicating that the GSR amplitude was circularly coupled with the EEG ISA phase (Figure 1D top). In other words, although the GRS maximum and minimum phase are independent parameters, they occur with a difference of nearly π. Using d-prime for quantifying discriminability and a *t*-test (*p* < 0.05) for statistical testing, we compared the distributions of the GSR amplitudes between the two indexed EEG ISA phases (Figure 1D bottom). The d-prime was calculated in order to compare between the GSR amplitudes at the EEG ISA phase with the maximum median GSR amplitude (peak) and those at the EEG ISA phase with the minimum median GSR amplitude (trough). Since the fact that the two indexed EEG ISA phases (peak and trough) were nearly π indicates that GSR value is circularly oscillated with in EEG ISA cycle, this d-prime calculation suggests the significant correlation between EEG ISA and GSR. The statistically significant coupling of the EEG ISA phase and the GSR amplitude (*t*-test, *p* < 0.05) suggested that the brain’s ISA phase correlates with the amplitude of the GSR.

**FIGURE 2:**
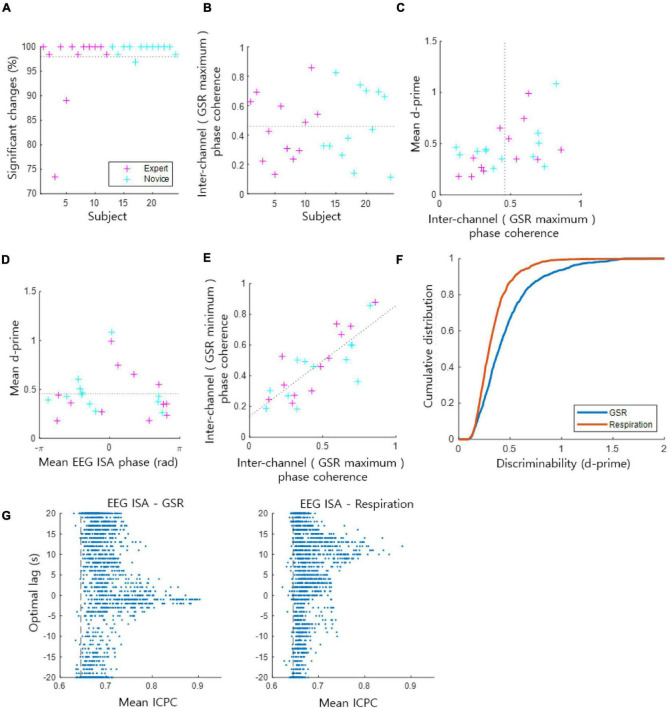
Coupling of the EEG ISA phase and the GSR amplitude. **(A)** The ratio of significant channels of the phase–amplitude coupling. The dotted horizontal line indicates the average for all subjects from the expert and novice meditator groups. **(B)** Inter-channel phase coherence (ICPC) at the phase for which the GSR magnitude is maximal in each subject. This represents the coherence of the GSR peak phases across channels. The dotted horizontal line indicates the average for all subjects. **(C)** Mean ICPC and mean d-prime for each subject. The dotted vertical line indicates the average ICPC for all subjects. **(D)** Mean EEG ISA phase and mean d-prime for each subject. The dotted horizontal line indicates the average of the mean d-prime values. **(E)** Inter-channel phase coherence based on the maximum GSR phase vs. the minimum GSR phase. Dotted line indicates the linear regression fit. **(F)** Comparison of the cumulative probability distribution of d-prime between the GSR and respiration. A higher d-prime value indicates stronger coupling between the EEG ISA phase and the GSR/respiration amplitude. **(G)** Inter-channel phase coherence (ICPC) between EEG ISA and GSR (left)/respiration (right) signals. Each dot indicates data of a channel of a subject. Vertical axis indicate the optimal lag of GSR/respiration signals. Vertical dashed lines indicate the 95% level of mean ICPCs of randomized phases.

Overall, 97.98 ± 5.71% [the mean ± the standard deviation of (the percentage of statistically significant channels in each subject) over all subjects] of the EEG channels across subjects exhibited statistically significant coupling of the EEG ISA phase with the GSR amplitude [*t*-test, *p* < 0.05, the population size was the number of indexed EEG ISA phases (phase at GSR maximum/minimum) by 1 Hz sampling in each channel and subject] (Figure 2A). From these significantly coupled channels, we assessed how the EEG ISA phase showing the maximum GSR was distributed over channels in each subject. This was quantified by ICPC, which ranges between 0 and 1. ICPC values varied substantially across subjects with a mean of 0.46 ± 0.23 (Figure 2B), indicating that the ICPC of individuals was widely distributed but centered at an intermediate value. There was no significant difference in ICPC between the expert and novice meditation groups (*t*-test, *p* > 0.87). The high-ICPC group, which included the subjects with an ICPC greater than the median ICPC, tended to exhibit high d-prime values (*t*-test, *p* < 0.05); high d-prime values suggest that the brain ISA phase strongly correlated with the amplitude of the GSR (Figure 2C). This demonstrates that the degree to which the EEG ISA and GSR are correlated is related to the inter-channel consistency in the correlation manner.

Monto et al. (2008) reported the grand average of subjects, so behavioral performance was maximal at a certain EEG ISA phase. In contrast, de Goede and van Putten (2019) reported individual differences in EEG ISA phases with maximum behavioral performance. In the present study, we evaluated the individual differences in the EEG ISA phase with the maximum GSR amplitude among subjects. We observed that the mean (of channels) phase exhibiting the maximum GSR varied from subject to subject (Figure 2D). Notably, the subjects who showed a mean phase with the maximum GSR near 0 tended to exhibit high d-prime values; high d-prime values suggest that the ISA phase strongly correlated with the amplitude of the GSR (Figure 2D). The mean d-prime was 0.45 ± 0.23 [the mean ± the standard deviation of (the mean of d-prime values across channels in each subject) overall subjects] (Figures 2C,D). The coherence of the maximum GSR phase across channels and the coherence of the minimum GSR phase across channels were proportional (Figure 2E, r-square = 0.64), suggesting that this coherence calculation based on the maximum GSR is similar to that based on the minimum GSR.

Lastly, we evaluated the coupling of the EEG ISA phase with the GSR in comparison with that of respiration, since 0.1-Hz EEG signals have been shown to be strongly correlated with respiration (Karavaev et al., 2018). We verified that the ISA phase was coupled with the GSR amplitude more strongly than respiration did by showing that the d-prime values of the GSR amplitude coupling were greater than those of the respiration coupling (*t*-test, *p* < 10^−48^, the population size was the sum of the number of statistically significant channels overall subjects) (Figure 2F). We performed an additional analysis of ICPC between EEG ISA and GSR/Respiration signals. To find optimal time lags between EEG ISA and GSR/Respiration signals, the lag with the highest ICPC value and the ICPC value at that lag were recorded while shifting the time of GSR/Respiration signals. For these ICPCs, 98.18% of GSR and 97.40% of Respiration were significantly larger than ICPC of randomized signals (permutation test, *p* < 0.05). Distribution of optimal lags was delayed more in Respiration than in GSR (Figure 2G). The optimal time lag with high ICPCs (ICPC > 0.75) between GSR and EEG ISA turned out to be −0.25 ± 3.03 s (nearly no delay; synchronized) and that between Respiration and EEG ISA was 10.94 ± 1.99 s (large delay). It reflects that EEG ISA responded faster to changes in arousal levels than respiration but as fast as GSR.

### Stronger Coupling Between the Infraslow Activity and the Galvanic Skin Response Is Associated With Stronger Modulation of Individual Electroencephalogram Infraslow Activity During Meditation

We investigated whether individual differences in the coupling the ISA phase with the GSR amplitude could be associated with individual EEG ISA characteristics. Among the many possible EEG ISA characteristics, we tested individual EEG ISA modulations during meditation and their relationship to coupling of the GSR. We divided the subjects into two groups according to the channel-average d-prime values in each subject: a high d-prime group (*n* = 12) and a low d-prime group (*n* = 12). The high d-prime group included subjects whose ISA phase was coupled with the amplitude of the GSR more strongly than that of any of the subjects in the low d-prime group. For data analysis and statistical testing, the sample size was set to 768 = (number of subjects = 12) × (number of channels = 64) in each group.

**FIGURE 3:**
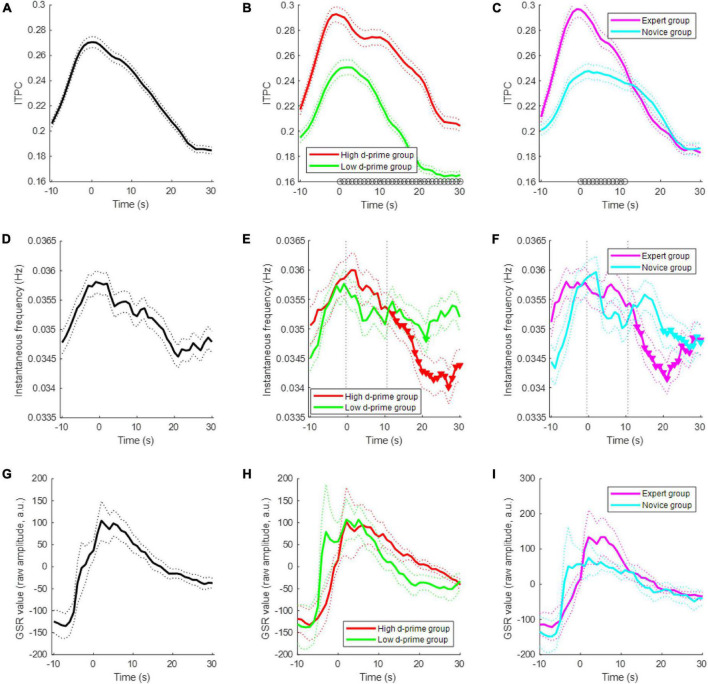
Electroencephalogram ISA modulation during meditation. **(A)** Changes of inter-trial phase coherence (ITPC) over time during the first 30-s period of meditation for all subjects. Each population size was set to 768 = (number of subjects = 12) × (number of channels = 64) for each time-point. A sample of the population is an ITPC value at a particular time-point, channel, and subject. The dotted line indicates the standard error of the mean. **(B)** ITPCs of the high d-prime group and low d-prime group. The black circles at each time-point indicate a statistically significant difference between groups (*t*-test, *p* < 0.05). Panel **(C)** similar to panel **(B)**, but showing the ITPCs of the expert and novice groups. **(D)** Changes in the instantaneous EEG ISA frequency for all subjects. The population size was set to 768 = (number of subjects = 12) × (number of channels = 64) for each time-point. A sample of the population is the median of instantaneous frequencies at a particular time-point, channel, and subject. **(E)** Instantaneous frequency changes of the high d-prime group and the low d-prime group. The inverted triangles indicate statistically significant decreases compared to the first 10 s. Panel **(F)** similar to panel **(E)**, but showing the instantaneous frequency changes of the expert and novice groups. **(G)** Changes of GSR for all subjects. Bold line indicates the mean and dotted lines indicate the standard error of the mean. **(H)** GSRs of the high d-prime group and low d-prime group. Panel **(I)** similar to panel **(H)**, but showing the GSRs of the expert and novice groups.

When a subject started meditating, the subject modulated their EEG ISA phase; the modulation of the EEG ISA phase indicates that the probability of a specific phase occurring at a specific point in time was increased. This ISA phase modulation was quantified by ITPC, which ranges between 0 and 1. We found that approximately 40% of the channels showed significant ITPC values (permutation test, *p* < 0.05) at the beginning of meditation, and this percentage of significant channels gradually decreased over time (Figure 3A). The ITPC of the high d-prime group was significantly higher than that of the low d-prime group during the period from meditation onset to 30 s after onset (*t*-test, *p* < 0.05) (Figure 3B). A comparison between the expert and novice groups also showed similar results, but significant differences persisted only for the first 11 s (*t*-test, *p* < 0.05) (Figure 3C).

The EEG infraslow frequency decreased slightly during meditation. In particular, the infraslow frequency was significantly decreased in the high d-prime group over time, whereas there was no significant decrease in the low d-prime group (*t*-test, *p* < 0.05) (Figure 3E). Similar results were found in the expert and novice groups, but the decrease and maintenance were less consistent (*t*-test, *p* < 0.05) (Figure 3F). The GSR value also decreases as meditation begins (Figures 3G–I).

## Discussion

Monto et al. (2008) showed that the EEG ISA phase is correlated with behavioral performance. However, it is unclear how the brain’s ISA phase can correlate with behavioral performance. The present study filled this gap by showing that the EEG ISA phase correlates with the GSR amplitude, which represents the arousal level; the arousal level is known to modulate behavioral performance. Arousal modulates behavioral performance and the level of arousal was observed both in GSR and EEG ISA. Furthermore, we analyzed whether meditation could modulate the EEG ISA and how the degree of modulation is related to the coupling between EEG ISA and arousal. We showed that subjects whose ISA phase was coupled with the GSR amplitude more strongly also modulated the EEG ISA more strongly during meditation, suggesting an intimate relationship between arousal level correlation with the brain ISA and the modulation of EEG ISA. Our results suggest that the arousal level may relate to the correlation of behavioral performance with the brain ISA.

Both the EEG ISA (Figure 1A) and GSR (Figure 1B) showed log–log linearity without clear peaks, which could be reminiscent of 1/f noise. However, this observation could also be made by the fact that EEG ISA and the GSR are quasi-periodic rather than 1/f noise (Palva and Palva, 2012, 2018). The result of coupling between the EEG ISA phase and the GSR amplitude might support this speculation of quasi-periodic EEG ISA.

Other types of phase–amplitude coupling between EEG and the GSR have been reported in previous studies (Kroupi et al., 2013), where coupling between the phase of the GSR and the amplitude of higher-frequency EEG activity (>1 Hz) was demonstrated. This is contrast to the phase-amplitude relationship in our case, which showed coupling between the phase of EEG ISA and the amplitude of the GSR; this has not been reported previously, to the best of our knowledge.

We observed that the EEG ISA frequency decreased over time after the onset of meditation, especially in the high d-prime group or in the expert meditation group (Figure 3). Although it is unclear why the EEG ISA frequency decreased during meditation, we speculate that it might be related to physiological changes during meditation, since EEG ISA correlates with respiration in addition to the GSR. Further investigations are required to investigate a possible relationship between physiological changes and EEG ISA during meditation; this would be helpful in understanding how to utilize EEG ISA for assessing the effects of meditation.

It is known that EEG ISA during human sleep correlates with the activity of non-specific thalamic nuclei associated with arousal (Picchioni et al., 2011). The phase of the EEG ISA in rodents during sleep is known to be associated with the level of arousal (Lecci et al., 2017). These results are consistent with the results of the present study. These suggest that EEG ISA could be associated with arousal in both humans and rodents, both sleep and non-sleep states.

There are several limitations to the present study. First, we did not compare EEG ISA and the GSR directly to behavioral performance data, because the dataset analyzed in this study does not contain behavioral performance data. We will conduct a follow-up study to confirm the arousal-related correlation between behavioral performance and ISA with new experiments. Second, we only used d-prime for the quantification of phase–amplitude coupling as opposed to using modulation indices, as in other phase–amplitude coupling analyzes, because we posited that d-prime might provide a more familiar and intuitive measure. Third, we investigated the relationships between the EEG ISA phase and the GSR amplitude specific to the meditation task in this study. However, it would be interesting to determine whether this coupling would also be present in other types of mental tasks.

In summary, we proposed that the correlation of behavioral performance with the ISA phase would be mediated by the modulation of arousal and showed that the arousal level (as measured by the GSR amplitude) was indeed related to the ISA phase. This finding may offer a new account of how the brain’s ISA modulates behavioral performance. We envision that EEG ISA might be used together with the GSR and other physiological signals for biofeedback training in neurological disorders.

## Data Availability Statement

The original contributions presented in the study are included in the article/Supplementary Material, further inquiries can be directed to the corresponding author.

## Ethics Statement

Ethical review and approval was not required for the study on human participants in accordance with the local legislation and institutional requirements. Written informed consent for participation was not required for this study in accordance with the national legislation and the institutional requirements.

## Author Contributions

DS: conceptualization, methodology, software, validation, formal analysis, investigation, resources, data curation, writing – original draft, writing – review and editing, visualization, supervision, and project administration. S-PK: writing – original draft, writing – review and editing, visualization, supervision, project administration, and funding acquisition. Both authors contributed to the article and approved the submitted version.

## Conflict of Interest

The authors declare that the research was conducted in the absence of any commercial or financial relationships that could be construed as a potential conflict of interest.

## Publisher’s Note

All claims expressed in this article are solely those of the authors and do not necessarily represent those of their affiliated organizations, or those of the publisher, the editors and the reviewers. Any product that may be evaluated in this article, or claim that may be made by its manufacturer, is not guaranteed or endorsed by the publisher.

## Footnotes

1
https://www.biosemi.com/activetwo_full_specs.htm
