# Supplementary material for: Brain Infraslow Activity Correlates With Arousal Levels
Source: Front Neurosci. 2022 Feb 25;16:765585. doi: 10.3389/fnins.2022.765585 (PMC8914100; doi:10.3389/fnins.2022.765585)
Supplement: Supplementary file 1 [file Data_Sheet_1.docx]

*Supplementary Material*

*of*

*Brain infraslow activity correlates with arousal levels*

# Spatial distributions of EEG ISA phases during meditation among subject groups

The analysis here aims to show that a specific EEG ISA phase is predominantly present when meditation is initiated. We investigated the differences in the EEG ISA phase, the phase of the oscillation cycle, distributions over the scalp for the first 30 s of meditation between the subject groups described above (high vs. low d-prime groups; or expert vs. novice meditation groups). These phase distributions are dependent on the phase reset at the onset of meditation and the shape of the EEG ISA waveform during meditation. Each of the four groups showed a characteristic phase distribution. In each of the groups, the ISA phases in the occipital region tended to be distributed mainly in the falling phase (approximately $\pi/2$ rad). In the high d-prime group, the ISA phases in the superior region were distributed in the rising phase (around $-\pi/2$ rad), while the ISA phases in the inferior region were distributed in the falling phase (Fig S1A). In the low d-prime group, the ISA phases were distributed in either the early or late falling phase; that is, at the lowest amplitude of EEG ISA (Fig S1A). In the expert group, the ISA phases in the superior region were distributed in the rising phase, while the ISA phases in the inferior region were distributed in the phase at the lowest amplitude of the EEG ISA (Fig S1B). In the novice group, the ISA phases tended to be distributed in the rising phase in regions other than the occipital region (Fig S1B). Since the EEG ISA phase distribution of 30 seconds after the start of meditation and 30 seconds before the start of meditation is different, it can be seen that the EEG ISA phase is reset when the meditation starts (Fig S1C and S1D).


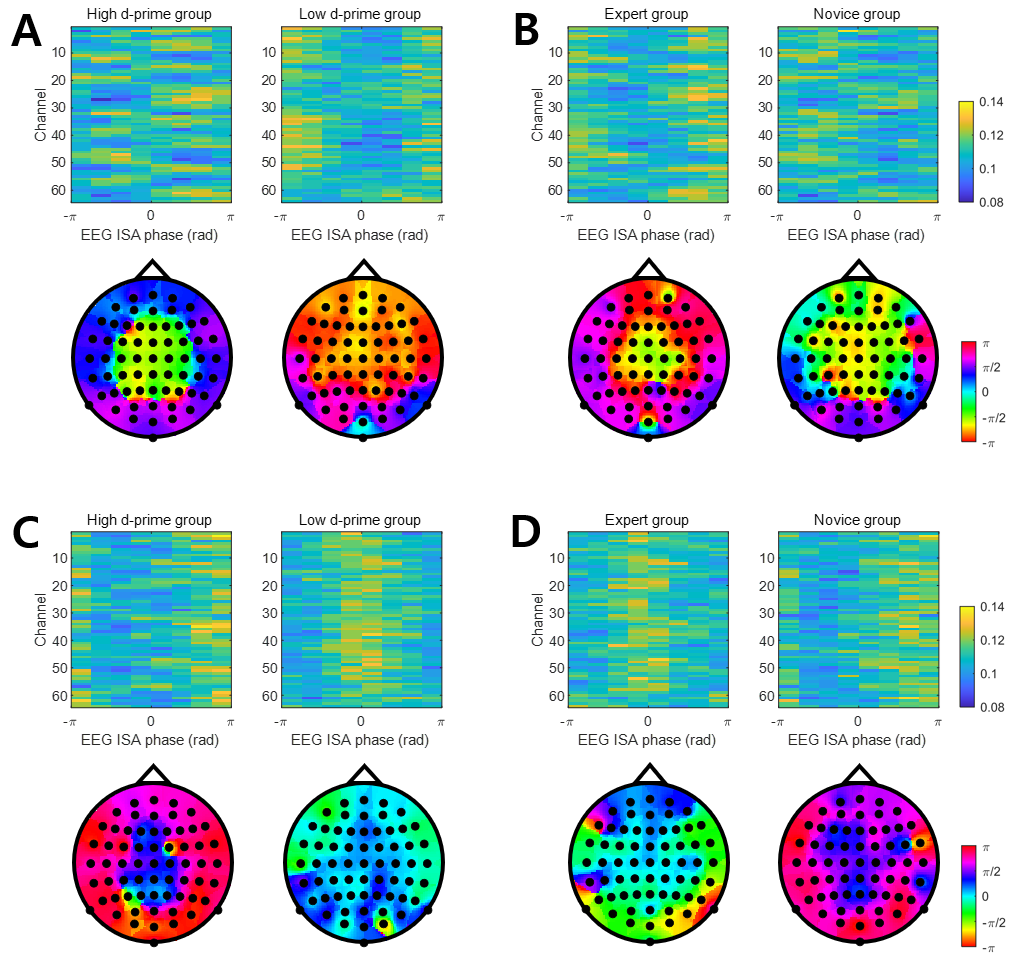


**Supplementary Figure 1. Distribution of EEG ISA phases during meditation.** (A) (top) Distribution of the EEG ISA phase during the first 30 s of meditation for each channel of the high (left) and low (right) d-prime groups. Note that the EEG ISA phases are not evenly distributed in this period. (bottom) Scalp topography map of the ISA phases for the channels of the high (left) and low (right) d-prime groups. (B) Similar to (A) but showing the expert (left) and novice (right) meditation groups. (C) Similar to (A) but distribution of the EEG ISA phase during the last 30 s of meditation. (D) Similar to (B) but distribution of the EEG ISA phase during the last 30 s of meditation.
